# Enhanced complex network influential node detection through the integration of entropy and degree metrics with node distance

**DOI:** 10.1038/s41598-025-15968-9

**Published:** 2025-08-25

**Authors:** Ramya D. Shetty, Rashmi M., Khyathi Rajesh Shetty, Manoj T.

**Affiliations:** 1 Department of Information and Communication Technology, Manipal Institute of Technology, Manipal Academy of Higher Education, Manipal, 576104 Karnataka India; 2 Department of Data Science and Computer Applications, Manipal Institute of Technology, Manipal Academy of Higher Education, Manipal, 576104 Karnataka India; 3 Department of AI & DS, NMAM Institute of Technology, Nitte, India; 4 Department of Computer Science and Engineering, Manipal Institute of Technology, Manipal Academy of Higher Education, Manipal, 576104 Karnataka India

**Keywords:** Complex network, Influential node, K-shell, Node entropy, Computer science, Information technology

## Abstract

Complex networks play a vital role in various real-world systems, including marketing, information dissemination, transportation, biological systems, and epidemic modeling. Identifying influential nodes within these networks is essential for optimizing spreading processes, controlling rumors, and preventing disease outbreaks. However, existing state-of-the-art methods for identifying influential nodes face notable limitations. For instance, Degree Centrality (DC) measures fail to account for global information, the K-shell method does not assign a unique ranking to nodes, and global measures are often computationally intensive. To overcome these challenges, this paper proposes a novel approach called Entropy Degree Distance Combination (EDDC), which integrates both local and global measures, such as degree, entropy, and distance. This approach incorporates local structure information by using entropy as a local metric and enhances the understanding of the overall graph structure by including path information as part of the global measure. This innovative method makes a substantial contribution to various applications, including virus spread modeling, viral marketing etc. The proposed approach is evaluated on six different benchmark datasets using well-known evaluation metrics and proved its efficiency.

## Introduction

Numerous real-world systems can be conceptualized as distinct complex networks^1^, such as marketing networks^2^, information dissemination networks^3^, urban transportation networks^4^, protein interaction networks^5^, epidemic transmission networks^6,7^, and many others. With the advancement in graph theory, research on complex networks is a more alluring topic in recent years due to its diverse application in social, chemical, and biological domains^8–10^. The nodes and edges are two primary components in complex networks^11^, representing the entities (such as humans, organizations, etc.) and the interactions among entities, respectively. The nodes, and edges play diverse roles in network structure and also its function^12^. According to^8^, spreading processes are basic and pervasive in many fields. They are crucial for a variety of purposes, such as the spread of ideas and news, the growth of social movements, the occurrence of disease outbreaks, and the promotion of products. Influential spreaders are nodes that contribute significantly to the spreading process in such complex networks.

Critical nodes have a completely different impact on the operation and structure of the network than other nodes. The enhancement or failure of critical nodes will result in a major change in the functioning of the network^13^. So, in recent research domain, a number of fields, including viral marketing, rumor analysis, infectious disease prevention, spreading dynamics, and evidence theory, have shown interest in the identification of vital nodes in networks^14–20^. For example, identifying influential entities in commercial networks will accelerate the spreading of marketing-related information and, in turn, will lead to a favorable income. Locating vital spreaders in information propagation networks will efficiently control the flow of rumors and lead to a healthy environment in society. Similarly, identifying vital nodes in epidemic models will significantly prevent the spreading of dangerous diseases among people.

The complex network research community proposed numerous centrality measures to identify crucial network nodes^21^. Most of the classical centrality include Degree Centrality (DC)^22^, Closeness Centrality (CC)^22^, Betweenness Centrality (BC)^23^, K-shell (KS) method^24^, Eigen-Vector Centrality (EC)^25^, and so on. The DC of a node is decided by the number of direct neighbors it has. According to DC, higher DC signifies that the node is connected to more neighbors and therefore is deemed more influential. However, this metric solely focuses on the node’s local connectivity and does not account for the broader structural information or the influence of its neighbors in the overall network.

The literature contains numerous examples of additional centrality measures that have been developed to address the weaknesses of traditional approaches by quantifying the significance of the node using the global structure of the network. For instance, the CC and BC measures rely on global information that is computationally expensive. These two methods are based on the concept of a shortest path between nodes^13^. CC evaluates the significance of a node by calculating the shortest distance between the node in question and all other nodes in the network. In contrast, BC evaluates the part of shortest paths in the whole complex network in which the respective node under consideration is a participant.

EC assesses the importance of a node by evaluating the significance of its neighborhood and the network as a whole. Consequently, EC states that a node has greater influence if its neighbors have greater influence. According to the K-shell approach^24^, the nodes in the center of the network are more important than others. K-shell, however, can produce the same value for many nodes in some networks, which can lead to confusion^26^.

Recently, research on complex networks to identify vital nodes, extending and improving upon the classical algorithms discussed earlier using local information and global structure, is a very alluring topic. K-shell decomposition and DC are among the most extensively studied methods for interpreting global and local network information. Due to their simplicity, these approaches have been widely adopted across networks of varying sizes. However, neither KS nor DC can reliably determine the relative importance of network nodes^27^. The Gravity Centrality (GC) measure was proposed by^28^ to determine the impact of a node by analyzing the K-shell values of the nodes and their minimal distances.

The importance of a node is quantified by Local-Global Centrality (LGC)^29^, which utilizes local and global information from DC and the shortest path, respectively. A K-shell and Structural Hole (SH) combination-based approach^30^ enhances the efficacy of K-shell by incorporating SH. Authors in^20^ introduced the Extended Clustering Coefficient Local Global Centrality (ECLGC) measure, which integrates the CLC with LGC. This measure is designed to identify influential nodes by focusing on both local and global data. As a consequence, the original structural information of the network may be significantly compromised when the nodes are sampled. A local structure entropy based method is proposed in^31^. Authors in^32^ proposed an approach for identifying influential nodes using gravity model-based H-index. In addition to more effectively identifying nodes with lesser K-shell values that bridge various network segments, this measure considers the role of adjacent nodes, path details, and node positions within the network.

Several approaches, for example^33–35^, based on the gravity model, are proposed in the existing literature to identify influential nodes. These methods benefit from node interaction details obtained through gravity-based models. In addition, several models proposed in the literature combine the K-shell with other structural properties of the complex network to improve the performance in vital node identification. Some of these methods, such as^16,36–39^, investigate advancements in the K-shell algorithm to identify influential nodes. They introduce enhancements, including the integration of K-shell with PageRank, the incorporation of iterative factors, and the adaptation of the system for community-based networks. These methods enhance the ranking of critical nodes, illustrating the algorithm’s adaptability and efficacy in a variety of scenarios by addressing challenges such as cascading failures and optimizing influence propagation.

Existing approaches to recognize influential nodes in complex networks face several significant challenges. DC exclusively concentrates on local connectivity, disregarding the global structural context and the influence of neighboring nodes. CC and BC are computationally intensive due to their reliance on shortest path computations, which restricts their scalability to more extensive networks, despite the fact that they leverage global structural information. Frequently, the K-shell decomposition process generates identical scores for multiple nodes, which leads to ambiguity in the ranking of their influence. Despite the fact that EC takes into account the significance of neighboring nodes, it is predicated on the assumption that influential neighbors naturally increase a node’s significance. However, this assumption may not be universally applicable across an array of network types. GC relies heavily on specific structural characteristics, such as K-shell values and minimum distances, reducing adaptability to different networks. LGC struggles to integrate local and global metrics effectively, leading to a less comprehensive assessment of node importance. Similarly, the ECLGC approach suffers from the loss of critical structural information due to node sampling, undermining its accuracy and effectiveness across various network applications. These limitations emphasize the need for a more robust and integrative approach to identify influential nodes accurately. In this paper, a novel measure of Entropy Degree Distance Combination (EDDC) is proposed to address these challenges, and its unique characteristics are as follows:

- The proposed approach incorporates three distinct measures: degree, entropy, and distance. This integration helps to identify influential nodes across various network structures.
- The proposed approach resolves the monotonicity ranking issue by assigning a unique ranking to each node, outperforming other recent methods.
- By utilizing entropy as a local measure, we have demonstrated the significance of Shannon’s entropy in gaining a deeper understanding of local influence, which plays a crucial role in formulating the proposed approach.

## Preliminaries

The literature suggests a variety of approaches to identify influential spreaders in intricate networks. In this section, we emphasize several fundamental methods that served as the impetus for the development of a novel centrality measure to identify influential nodes. In mathematical notations, the complex networks are represented as a graph $G = (V(G), E(G))$ with $N=|V(G)|$ nodes and $E=|E(G)|$ edges. Where, $V(G) = \{v_{1}, v_{2},..., v_{N}\}$ and $E(G) = \{e_{1}, e_{2},..., e_{E}\}$ represents the node and edge set respectively. The network is represented as adjacency matrix, $A_{G}=(a_{v_{i}v_{j}})_{N \times N}$. Where the entry $a_{v_{i}v_{j}}$ represents whether node $v_{i}$ and $v_{j}$ are directly connected (value 1) or not (value 0). Further, $\Gamma (v_{i})$, and $dist(v_{i},v_{j})$ represents the set of single hop neighborhood of node $v_{i}$, and smaller path distance between pair of nodes ($v_{i}$, $v_{j}$). The proposed work targets undirected and unweighted networks with *N* nodes. The following is a description of existing node centrality measures explored in the literature to identify vital nodes based on local, global, and hybrid measures.

### Degree Centrality (DC)

DC^22^ is a measure that assesses the direct influence of a node among other nodes in the network utilizing only local information. So, the node with a higher DC indicates more influence. Mathematically, DC^32^ is represented as shown in Eq. (1).

1 $$\begin{aligned} DC(v_{i})= |\Gamma (v_{i})| \end{aligned}$$

Where, $DC(v_{i})$, $\left| \Gamma (v_{i}) \right|$, denotes the DC of node $v_{i}$, and count of one-hop neighbors or degree of a node, respectively.

### Betweenness Centrality (BC)

BC^23^ is a metric that assess the influence of a node by analyzing global data. A node $v_{i}$’s BC is computed by the percentage of shorter paths that pass through it. Thus, the node with the highest BC is easily accessible to other nodes in the network and exerts a significant influence on them. Mathematically, BC is defined as shown in Eq. (2).

2 $$\begin{aligned} BC(v_{i})= \sum _{j\ne i\ne k\in V(G)}\frac{SP_{v_{j}v_{k}}(v_{i})}{SP_{v_{j}v_{k}}}, \end{aligned}$$

Where, $BC(v_{i})$, $SP_{v_{j}v_{k}}(v_{i})$, and $SP_{v_{j}v_{k}}$ represents BC of node $v_{i}$, shortest paths between nodes $v_{j}$, and $v_{k}$ which passes through node $v_{i}$, and shortest paths between nodes $v_{j}$, and $v_{k}$ respectively.

### Closeness Centrality (CC)

CC^22^ of a node measures the influence utilizing the global data. According to CC, node closer to remaining set of nodes is more prominent node in the network. Finally, CC of node $v_{i}$ is determined by analyzing the shortest path distances between node $v_{i}$ and remaining nodes in the network, mathematically expressed as given in Eq. (3).

3 $$\begin{aligned} CC(v_{i})= \frac{N-1}{\sum _{j \in V(G)}dist(v_{i},v_{j})}, \end{aligned}$$

### Clustering Coefficient Centrality (CLC)

CLC^40^ measures the level of involvement of a node in tightly connected clusters in the graph. It is computed as the ratio between the count of edges between the neighbors of the node under consideration and the total number of possible such edges. Eq. (4) is the mathematical representation of CLC of node $v_{i}$.

4 $$\begin{aligned} CLC(v_{i})= \frac{2 \times |E(i)|}{|\Gamma (v_{i})| \times |(\Gamma (v_{i})|-1) }, \end{aligned}$$

Where, $|\Gamma (v_{i})|$ represents the count adjacent nodes of node $v_{i}$. Whereas |*E*(*i*)| represents the count of edges present between nodes in the set $\Gamma (v_{i})$. As the proposed work utilizes only an undirected graph, the constant 2 is used in the numerator of the equation.

### Isolating Centrality (ISC)

ISC^41^ measure aims to identify critical nodes that divide the network into distinct components. It is determined using two factors: the degree of the node and the minimum degree among its neighboring nodes. Mathematically, it is expressed as shown in Eq. (5). Based on this, a high-degree node is not influential if its neighbor’s degree is less than a threshold.

5 $$\begin{aligned} ISC(v_{i}) = \left| N(v_{i}) \cap \text {Deg}_{\delta } \right| \times \left| \Gamma (v_{i})\right| , \end{aligned}$$

where $v_{i}$ is the node under consideration. The first component $\left| N(v_{i}) \cap \text {Deg}_{\delta } \right|$ in the equation is the isolating coefficient of the node and is defined by its neighbor nodes with a degree of $\delta$. The second component $\left| \Gamma (v_{i})\right|$ is the degree of the node under consideration.

### Local-Global Centrality (LGC)

Recently, identifying vital nodes by fusion of both local and global data in the network is more trending among researchers. As the size of complex networks is increasing rapidly, the connectivity information of the node at local and global levels is essential to decide its role in the whole network. One such measure is the LGC^29^ of a node considering both local and global influence of the node is shown in Eq. (6).

6 $$\begin{aligned} LGC(v_{i})= \frac{|\Gamma (v_{i})|}{N} \times \sum _{i \ne j}\frac{\sqrt{|\Gamma (v_{j})|}}{dist(v_{i},v_{j})} \end{aligned}$$

Where, the first component of the equation $\frac{|\Gamma (v_{i})|}{N}$ is the local influence of node $v_{i}$. In which, $|\Gamma (v_{i})|$, *N* represents the degree of node $v_{i}$ and the count of nodes in the network, respectively. The second component $\sum _{i \ne j}\frac{\sqrt{|\Gamma (v_{j})|}}{dist(v_{i},v_{j})}$ quantifies the global influence of the node $v_{i}$.

### K-shell value

The K-shell value^42^ of node is computed as follows^24,43^: at first, eliminate all nodes with a degree $k=1$, and proceed until there are no more nodes with a degree of one. To all eliminated nodes assign $k=1$ as K-shell value. Next, iteratively repeat the process of removing nodes by increasing the degree threshold *k*. Process termination occurs when there are no additional nodes to be removed.

### Clustering Coefficient Local-Global Centrality (CLGC)

CLGC^20^ of a node is based on local and global influence. This approach computes the node’s CLC in 2 different levels to get local and global information. The CLGC of a node $v_{i}$ is denoted by Eq. (7).

7 $$\begin{aligned} CLGC(v_{i})= CLC(v_{i}) \times \sum _{i \ne j}\frac{\sqrt{CLC(v_{j})}}{dist(v_{i},v_{j})} \end{aligned}$$

Where, the first component CLC of node $v_{i}$ captures the local influence. Where as the second component $\sum _{i \ne j}\frac{\sqrt{CLC(v_{j})}}{dist(v_{i},v_{j})}$ is based on global information, where $CLC(v_{j})$ is the CLC of node $v_{j}$.

### Extended Clustering Coefficient Local Global Centrality (ECLGC)

ECLGC^20^ is another way of finding vital nodes combining CLC in different levels. Mathematically, it is expressed as Eq. (8).

8 $$\begin{aligned} ECLGC(v_{i})=\left[ CLC(v_{i}) + \sum _{j \in \Gamma (v_{i})}\frac{\sqrt{CLC(v_{j})}}{\left| \Gamma (v_{i})\right| } \right] + \left[ \sum _{i \ne j} \frac{ \sqrt{ CLC(v_{j}) + \sum _{k \in \Gamma (v_{j})}\frac{\sqrt{CLC(v_{k})}}{\left| \Gamma (v_{j})\right| } }}{dist(v_{i},v_{j})} \right] , \end{aligned}$$

Where, $CLC(v_{i})$, $CLC(v_{j})$, $CLC(v_{k})$ denotes CLC of nodes $v_{i}$, $v_{j}$, and $v_{k}$ respectively. The neighbors of nodes $v_{i}$, and $v_{j}$ denoted as $\Gamma (v_{i})$, and $\Gamma (v_{j})$, respectively.

## Proposed method

The proposed EDDC method aims to develop a generalized approach that integrates three key measures: degree, distance, and entropy influence scores. Combining these important metrics into a single formula allows the EDDC measure to identify influential nodes in the network effectively. Each component captures distinct yet complementary aspects of a node’s role in the network:

- **DC** captures local structural importance by measuring how well-connected a node is to its immediate neighbors. However, it lacks the ability to distinguish between nodes that have the same degree but differ in how information is distributed across their connections.
- **Entropy** compensates for this limitation by introducing local information diversity. Even among nodes with the same degree, those connected to nodes of varying importance or edge strength will exhibit different entropy values. This helps refine the influence measure by considering the variability in local interactions, rather than just their count.
- **Distance (shortest path)** introduces a global structural dimension. It enables the method to consider how far a node’s influence can potentially reach in the network, thus addressing the network-wide spread of influence. By inversely weighting the entropy contributions by distance, the method ensures that closer nodes contribute more to the influence score, reflecting the intuition that influence weakens over distance.

The behavior of entropy, whether it is considered a local or global measure, depends on the scope of the information utilized. Specifically, it varies based on whether the entropy is calculated using the data from a node’s immediate surroundings (local) or from the entire graph (global). In our study, we treat node entropy as a local measure because we calculate the entropy score for each node based solely on the information from its immediate neighbors. Shannon’s entropy^44^ measures the expected amount of information in an event and is expressed as follows:

9 $$\begin{aligned} e(v_i) = - \sum _{j \in \Gamma (v_{i})} p(v_j) \log _2 p(v_j). \end{aligned}$$

Where, $e(v_i)$ denote the entropy of node $v_i$, $p(v_j)$ refers to the probability value based on neighbor degree of node $v_{i}$ and its computation method is shown in Eq. (10). The $\sum _{j}$ is over all neighbors of node $v_{i}$, and $\log _2$ is the base-2 logarithm, which quantifies the information content of the node.

10 $$\begin{aligned} p(v_j) = \frac{deg(v_j)}{\sum _{k \in \Gamma (v_i)} deg(v_k)}. \end{aligned}$$

Where, $v_i$ denotes the node for which entropy is computed, $deg(v_j)$ is the degree of the neighbor node $v_j$ of $v_i$, and $\Gamma (v_i)$ represents set of immediate neighbors of node $v_i$. In addition to the entropy measure, the proposed method includes two critical factors: the degree and distance measures. The shortest path between node under consideration and the remaining nodes of the network is incorporated into the proposed method. The proposed method, called the Entropy Degree Distance Combination (EDDC), is expressed as follows:

11 $$\begin{aligned} EDDC(v_{i})= \frac{\left| \Gamma (v_i)\right| }{N} \times \sum _{ j \in V(G) \& i \ne j}\frac{\sqrt{e(v_{i}) + e(v_{j})}}{dist(v_{i},v_{j})} \end{aligned}$$

Where, $EDDC(v_{i})$, *N*, $\left| \Gamma (v_i)\right|$, $e(v_{i})$, $e(v_{j})$, and $dist(v_{i},v_{j})$ denotes EDDC of node $v_{i}$, total nodes in the network, number of neighbors of node $v_{i}$, entropy of node $v_{i}$, entropy of node $v_{j}$, and shortest distance between node $v_{i}$ and $v_{j}$ respectively. The formula initially computes a weighted sum of the entropies of neighboring nodes, adjusted by their distances, and then scales it by the node’s degree, normalized by node count $N$. This integrated measure aids in identifying influential nodes by considering local structural features (degree), informational content (entropy), and spatial relationships (distance) between nodes. It proves to be a powerful tool for network analysis, particularly in complex systems where proximity and information flow determine influence.

The proposed method is formulated to incorporate $e(v_i)$ within the summation to reflect the *intrinsic information diversity* of node $v_i$ as it interacts with other nodes across the network. This approach captures the idea that a node’s influence is not only shaped by the entropy of other nodes but also moderated by its own informational richness. Even when $dist(v_i, v_j)$ is large, the inverse distance weighting significantly reduces the contribution of distant nodes. Thus, while $e(v_i)$ appears in every term, its impact on distant contributions is naturally minimized by the denominator. This aligns with influence propagation models where a node’s structural diversity serves as a baseline factor in assessing its global influence.

The use of the square root aggregation was motivated by the need to avoid domination by large entropy values in densely connected regions. A linear sum $e(v_i)+e(v_j)$ tends to overemphasize high-entropy nodes, which may bias the ranking towards nodes in high-degree clusters. The radical formulation introduces a sublinear growth that moderates such effects, resulting in more balanced and robust rankings across networks with heterogeneous degree distributions (as observed in preliminary experiments). Conceptually, this means that as two diverse neighborhoods interact, their combined contribution increases more slowly because some of their information may overlap or repeat.

The Algorithmic representation of EDDC computation of all the nodes in the network and the entropy of a node calculation are shown in Algorithm 1 and 2, respectively.

**Algorithm 1:**
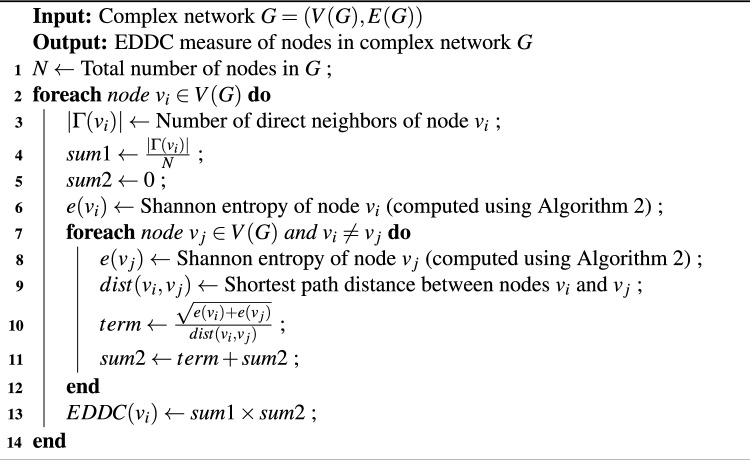
Compute EDDC for Nodes in a Complex Network.

**Algorithm 2:**
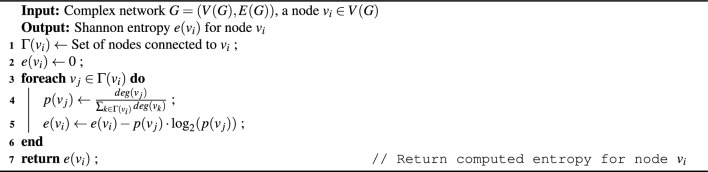
Compute Shannon Entropy $e(v_i)$ for a Node.

**Fig. 1:**
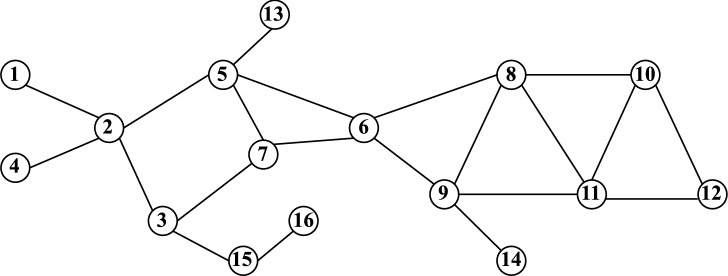
Toy network used in experimentation having 16 nodes and 21 edges.

### Computation steps

Here, we present a step-by-step calculation of our proposed approach. Our objective is to assess the influence score of every node in the network. For demonstration purposes, we focus specifically on calculating the influence score of node 2. The process begins with the computation of entropy using a small toy network (Fig. 1) as an example. Node 2 neighbors are node {1, 3, 4, 5}. The no. of degrees of those neighbor nodes are {1, 3, 1, 4} respectively. According to Eq. (10), the total no of edges = 1+3+1+4 = 9. Further, we need to calculate $p(e_1) = \frac{1}{9}$ = 0.111, $p(e_3) = \frac{3}{9}$ = 0.333, $p(e_4) = \frac{1}{9} = 0.111$, $p(e_5) = \frac{4}{9} = 0.444$.

In Step 2, by applying Eq. (9), we compute the entropy of node 2 by aggregating the individual scores from its neighboring nodes.

$$\begin{array} {l}e(2) = -[\frac{1}{9} log_{2}^{\frac{1}{9}} + \frac{3}{9} log_{2}^{\frac{3}{9}} + \frac{1}{9} log_{2}^{\frac{1}{9}} + \frac{4}{9} log_{2}^{\frac{4}{9}}] \\ \quad\quad = -[0.111 * -3.171 + 0.333 * -1.586 + 0.111 * -3.171 + 0.444 * -1.171] \\ e(2)= 1.752. \end{array}$$

Similarly, the entropy needs to be calculated for all the nodes. The computed entropy scores are as follows: $e(1) = 0$, $e(3) = 1.530$, $e(4) = 0$, $e(5) = 1.855$, $e(6) = 1.989$, $e(7) = 1.572$, $e(8) = 1.989$, $e(9) = 1.854$, $e(10) = 1.521$, $e(11) = 1.950$, $e(12) = 0.985$, $e(13) = 0$, $e(14) = 0$, $e(15) = 0.811$, and $e(16) = 0$. After calculating the entropy score, our objective is to determine the DC of node 2, which is equal to 4. With $N = 16$, the subsequent phase entails considering the computed quantity in conjunction with the distance measure, as defined by Eq. (11).

$$\begin{array} {l} EDDC(2) = \frac{{DC(2)}}{N} \times [\frac{{\sqrt {e(2) + e(1)} }}{{dist(2,1)}} + \frac{{\sqrt {e(2) + e(3)} }}{{dist(2,3)}} + \frac{{\sqrt {e(2) + e(4)} }}{{dist(2,4)}} + \frac{{\sqrt {e(2) + e(5)} }}{{dist(2,5)}} + \frac{{\sqrt {e(2) + e(6)} }}{{dist(2,6)}} \\ \quad \quad \quad \quad\quad\quad + \frac{{\sqrt {e(2) + e(7)} }}{{dist(2,7)}} + \frac{{\sqrt {e(2) + e(8)} }}{{dist(2,8)}} + \frac{{\sqrt {e(2) + e(9)} }}{{dist(2,9)}} + \frac{{\sqrt {e(2) + e(10)} }}{{dist(2,10)}} + \frac{{\sqrt {e(2) + e(11)} }}{{dist(2,11)}} + \frac{{\sqrt {e(2) + e(12)} }}{{dist(2,12)}} \\ \quad \quad \quad \quad\quad \quad + \frac{{\sqrt {e(2) + e(13)} }}{{dist(2,13)}} + \frac{{\sqrt {e(2) + e(14)} }}{{dist(2,14)}} + \frac{{\sqrt {e(2) + e(15)} }}{{dist(2,15)}} + \frac{{\sqrt {e(2) + e(16)} }}{{dist(2,16)}}]\\ \quad \quad \quad \quad\quad = \frac{4}{{16}} \times [\frac{{\sqrt {1.752 + 0} }}{1} + \frac{{\sqrt {1.752 + 1.530} }}{1} + \frac{{\sqrt {1.752 + 0} }}{1} + \frac{{\sqrt {1.752 + 1.855} }}{1} + \frac{{\sqrt {1.752 + 1.989} }}{2} \\ \quad \quad \quad \quad \quad \quad + \frac{{\sqrt {1.752 + 1.572} }}{2} + \frac{{\sqrt {1.752 + 1.989} }}{3} + \frac{{\sqrt {1.752 + 1.854} }}{3} + \frac{{\sqrt {1.752 + 1.525} }}{4} + \frac{{\sqrt {1.752 + 1.950} }}{4} \\ \quad \quad \quad \quad \quad \quad + \frac{{\sqrt {1.752 + 0.985} }}{5} + \frac{{\sqrt {1.752 + 0} }}{2} + \frac{{\sqrt {1.752 + 0} }}{4} + \frac{{\sqrt {1.752 + 0.8112} }}{2} + \frac{{\sqrt {1.752 + 0} }}{3}] = 13.265. \end{array}$$

## Experimental setup

Here, we started by providing dataset descriptions along with the evaluation metrics, such as Kendall’s correlation score. The experiments that were conducted to illustrate the efficacy of our algorithm are detailed in this section. First, we evaluated Kendall’s correlation between the SIR model and different comparison algorithms. Next, we analyzed the monotonicity ranking among the various algorithms. Finally, we calculate the percentage of improvement of proposed EDDC measure compared to the baseline methods.

### Datasets

To evaluate the efficacy of the proposed *EDDC* method, we analyzed six complex networks with distinct structural properties. Table 1 provides an overview of these networks, highlighting their varying sizes, where $\beta$ denotes the infection probability rate, and $\beta _{th}$ indicates the threshold for network infection probability.

### Discussion on scale-free and small-world networks

To investigate whether the given network exhibits scale-free characteristics, we analyzed its degree distribution using the methodology proposed by^45^. The degree distribution was fitted with a power-law model, and its plausibility was evaluated using the Kolmogorov-Smirnov (KS) statistic and a likelihood ratio test against alternative distributions (specifically the lognormal). Table 2 provides a detailed analysis of the scale-free properties across the various networks examined in our study.

The concept of small-world networks^40^, is characterized by a high CLC and a short average path length (L), where CLC is significantly higher than that of a comparable random network, and L is approximately similar. While the original methodology relies on comparison with random graphs to establish small-world behavior, in real-world network analysis, these properties can also be evaluated intrinsically. In our study, we adhered to the traditional approach by comparing the CLC and average path length of the given networks with those of corresponding random graphs with equal size and density. This allowed us to quantitatively assess the extent to which the networks exhibit small-world characteristics. Empirical evidence supporting the small-world properties of the networks is presented in Table 3. Based on the above observations, we conclude that the majority of the networks used in our experimental study exhibit both scale-free and small-world properties.

**Table 1:** Network structural properties.

Network	${{\varvec{E}}}$	${{\varvec{V}}}$	${{\varvec{\beta _{th}}}}$	${{\varvec{\beta }}}$
soc-wiki-vote^46,47^	2914	889	0.055	0.02
jazz^46,48^	2742	198	0.026	0.06
fb-pages-food^46,49^	2102	620	0.05	0.02
fb-pages-politician^46,49^	41729	5908	0.023	0.01
Conference^50^	17327	262	0.007	0.01
Contacts in a Workplace-1^51^	755	92	0.05	0.12

**Table 2:** Scale-free property analysis includes the estimated power-law exponent ($\alpha$), Kolmogorov-Smirnov (KS) statistic, log-likelihood ratio comparing power-law and lognormal fits (R), and the corresponding p-value.

Network	${{\varvec{\alpha }}}$	${{\varvec{KS}}}$	${{\varvec{R}}}$	${{\varvec{p}}}$	Scale-free network property
soc-wiki-vote	3.396	0.041	-0.009	0.912	holds
jazz	5.269	0.063	-0.255	0.663	holds
fb-pages-food	2.651	0.044	-1.605	0.268	holds
fb-pages-politician	3.245	0.023	-2.891	0.122	holds
Conference	7.028	0.106	-8.191	0.012	does not hold
Contacts in a Workplace-1	7.479	0.069	-2.010	0.344	holds

**Table 3:** Small-world Property Analysis includes the path length (L) and CLC of the six real-world networks are compared against those of random graphs having the same number of nodes and average degree per node^40^. The small-world phenomenon holds when $L_{actual} \ge L_{random}$ and ${CLC}_{actural} \ge {CLC}_{random}$.

Network	${{\varvec{L_{actual}}}}$	${{\varvec{L_{random}}}}$	${{\varvec{{CLC}_{actual}}}}$	${{\varvec{{CLC}_{random}}}}$	Small-world network Property
soc-wiki-vote	4.096	3.825	0.152	0.006	holds
jazz	2.235	1.876	0.617	0.138	holds
fb-pages-food	5.088	3.573	0.330	0.010	holds
fb-pages-politician	4.664	3.579	0.385	0.002	holds
Conference	1.497	1.493	0.686	0.507	holds
Contacts in a Workplace-1	1.964	1.865	0.426	0.175	holds

### Evaluation metrics

The performance of the proposed centrality measure to find influential nodes in complex networks is evaluated using the SIR model and Kendall’s correlation score.

- **SIR Model:** The SIR model^52^ is a compartmental model that has been widely used to analyze the dynamics of information transmission in complex networks. It is used to characterize the spread of infectious diseases within a population. The population is divided into three states: *S* (Susceptible) individuals who are at risk of infection; *I* (Infected) individuals who are infected and have the potential to transmit the disease to susceptible; and *R* (Recovered) individuals who have recovered and acquire immunity. Every node is initially in the *S* state, except the node that is initialized in the *I* state. The infectious node acts as the seed for information spread. The model progresses through interactions where infections spread until the system stabilizes with no further infections. In every time step, *I* state nodes spread the information to their *S* state neighbors with a probability $\beta$. Afterward, they are transferred to the *R* state with a rate of $\lambda$, where they gain immunity and can no longer be infected. In our experiments, the spreading probability $\beta$ is varied between 0.01 to 0.15. The recovery probability $\lambda$ and total iterations are set as 1 and 1000, respectively.
- **Kendalls correlation score:** Additionally, Kendall’s tau^53^, a widely used metric for evaluating the level of the correlation analysis between two sequences. For every one of the previously discussed approaches, we derive the final node ranking. The correlation analysis between a given topology-related ranking and the ranking determined by the nodes’ true spreading ability is subsequently assessed using Kendall’s tau rank correlation coefficient $\tau$. Let $X$ and $Y$ be two sequences of equal length. Two pairs of elements, $(x_i,y_i)$ and $(x_j,y_j)$, are picked from corresponding locations in the sequences. The counts of concordant and discordant pairs for the two sequences $X$ and $Y$ of length *n* are denoted by $N_c$ and $N_d$, respectively. Kendall’s tau ($\tau$) for these sequences is then expressed as:
  12 $$\begin{aligned} \tau =\frac{N_c - N_d}{0.5n(n-1)} \end{aligned}$$
  $\tau$ values vary from -1 to 1. Positive and negative correlation are denoted by $\tau > 0$ and $\tau < 0$, respectively. In other terms, the ranking is more precise when the $\tau$ value is higher. To validate the efficiency of the EDDC method, we initially implement the SIR model to determine the ranking of nodes according to their propagation capacity. Subsequently, we compute the node influence ranking sequence that was obtained using the proposed approach. Finally, we compare the two sequences using this metric.

## Results and discussions

The proposed EDDC and baseline methods, including BC, CC, CLC, LGC, ISC, CLGC, and ECLGC, have been compared to quantify, evaluate, and compare the influence of nodes across various complex networks. The study involved three types of experiments:

- Experiment 1: The SIR model and the Kendall $\tau$ correlation coefficient were employed to assess the efficacy of the proposed indexing method. The indexing method’s efficiency is denoted by the $\tau$ value.
- Experiment 2: This experiment assessed the uniqueness of node rankings, focusing on the monotonicity of rankings within the network for various indexing methods.
- Experiment 3: The ranking relationships between the proposed technique, EDDC, and various baseline measures were examined. This analysis evaluates the extent to which the *EDDC* technique enhances the current indexing methods. The performance gain metric is represented as $\eta \%$^54^.

### Analysis of the Kendall $\tau$ coefficient in the SIR model across various indexing methods

**Fig. 2:**
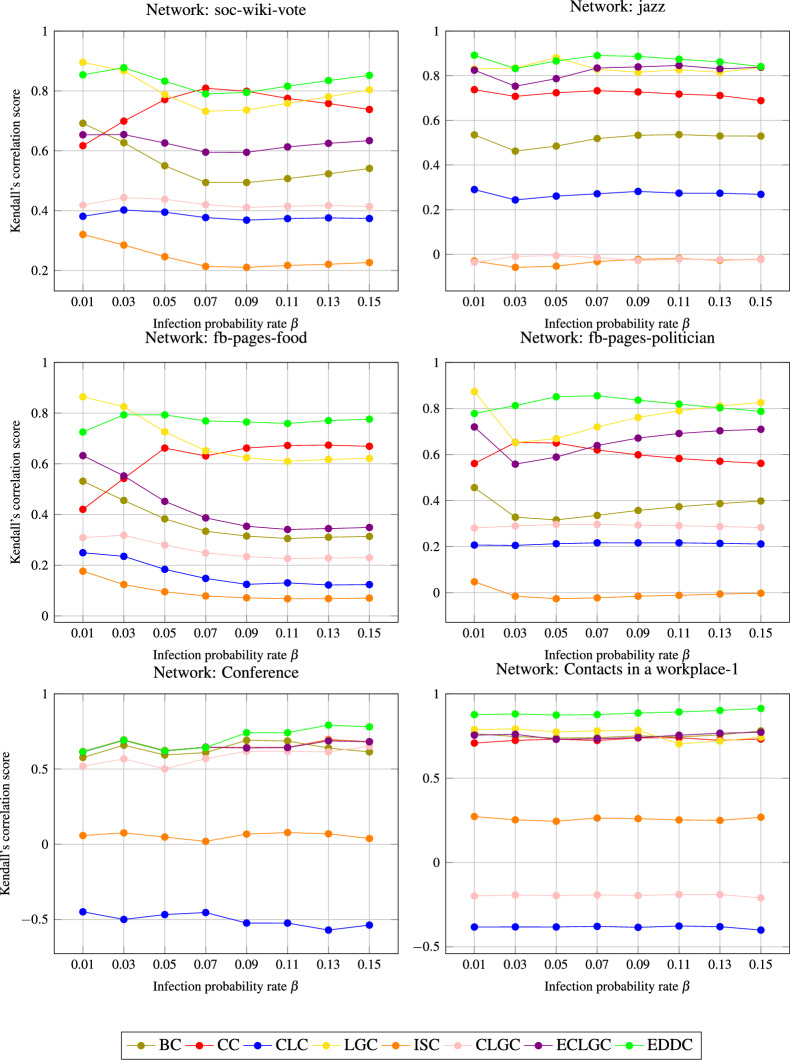
Kendall $\tau$ coefficient analysis between the SIR model and baseline methods at different infection probabilities $\beta$ across six networks.

To evaluate the rankings generated by different indexing methods, we employ the Kendall $\tau$ coefficient^24,8^, with the SIR model ranks serving as the reference baseline. Figure 2 compares six distinct complex networks. The X-axis represents the probability of infection $\beta$ (varies from 0.01 to 0.15), while Y-axis displays the $\tau$ values achieved by various indexing approaches against the SIR baseline. Notably, the *EDDC* method demonstrates superior performance compared to other techniques, especially in scenarios where $\beta$ exceeds the threshold $\beta _{th}$. For various complex networks, the global measures *BC*, *CC*, *ISC*, and *CLC* demonstrate poor performance, while the LGC method performs moderately. The more recent methods, CLGC and ECLGC, show a decline in performance compared to the proposed EDDC approach, which effectively outperforms all recent indexing measures.

Table 4 presents the comparison of the Kendall coefficient $\tau$ across different networks. To evaluate the consistency of the results, we determine the average Kendall value $\tau$ for scenarios where $\beta$ exceeds the threshold $\beta _{th}$, using Eq.(13).

13 $$\begin{aligned} \sigma (\tau )=\frac{\sum ^N_{i=1} \tau (\beta _{th}+0.01* i)}{N}. \ \end{aligned}$$

In this experiment, 15 iterations were conducted, with the value of $\beta$ increased by 0.01 in each iteration. The findings indicate that the *EDDC* method works effectively in both small- and large-scale networks. In addition, no tunable parameters were required to calculate the influence scores.

**Table 4:** The mean rank analysis correlation (Kendall’s $\tau$), represented as $\sigma$, for complex networks across various infection probability range.

Network	${\varvec{\sigma (\tau _{BC})}}$	${\varvec{\sigma (\tau _{CC})}}$	${\varvec{\sigma (\tau _{CLC})}}$	${\varvec{\sigma (\tau _{LGC})}}$	${\varvec{\sigma (\tau _{ISC})}}$	${\varvec{\sigma (\tau _{CLGC})}}$	${\varvec{\sigma (\tau _{ECLGC})}}$	${\varvec{\sigma (\tau _{EDDC})}}$
soc-wiki-vote	0.549	0.752	0.381	0.791	0.239	0.422	0.622	0.830
jazz	0.514	0.719	-0.270	0.835	-0.031	-0.017	0.816	0.868
fb-pages-food	0.364	0.687	0.160	0.688	0.092	0.256	0.419	0.771
fb-pages-politician	0.363	0.603	0.213	0.755	-0.009	0.291	0.656	0.778
Conference	0.633	0.661	-0.503	0.659	0.044	0.588	0.659	0.706
Contacts in a workplace-1	0.754	0.727	-0.387	0.762	0.259	-0.200	0.748	0.887

### Ranking uniqueness

Different baseline indexing methods and proposed measure are analyzed in this study to determine the unique ranking of the nodes. Monotonicity refers to the capacity to distinguish each and every node within the complex network. As described in^55^, monotonicity is expressed as $M_c(I)$, where *I* represents the ranking list produced by an indexing method, and is defined by the following formula:

14 $$\begin{aligned} M_c(I) =\Big [1-\frac{\sum ^N_{i\in I} N_i(N_i-1)}{N(N-1)}\Big ] ^2. \ \end{aligned}$$

In this context, *N* represents the number of nodes, while $N_i$ denotes the set of nodes sharing the same score *i*. The monotonicity measure, $M_c(I)$, takes values within the range [0, 1], where 0 indicates identical rankings, and 1 signifies completely distinct rankings. The monotonicity values for the eight indexing methods are provided in Table 5. The CLC approach demonstrates poor performance in assigning unique rankings to the nodes within the network. In contrast, the proposed EDDC method offers distinct rankings for all the datasets examined in this study, outperforming the other seven baseline indexing methods.

**Table 5:** Monotonicity ranking.

Network	${\varvec{M_c({BC})}}$	${\varvec{M_c({CC})}}$	${\varvec{M_c({CLC})}}$	${\varvec{M_c({LGC})}}$	${\varvec{M_c({ISC})}}$	${\varvec{M_c({CLGC})}}$	${\varvec{M_c({ECLGC})}}$	${\varvec{M_c({EDDC})}}$
soc-wiki-vote	0.876	0.998	0.623	0.999	0.099	0.638	0.993	0.999
jazz	0.988	0.987	0.991	0.999	0.002	0.997	0.999	0.999
fb-pages-food	0.835	0.997	0.746	0.998	0.070	0.778	0.978	0.999
fb-pages-politician	0.943	0.999	0.900	0.999	0.017	0.920	0.998	0.999
Conference	0.999	0.988	1.0	1.0	0.2005	1.0	1.0	1.0
Contacts in a workplace-1	0.943	0.969	0.991	0.954	0.069	1.0	1.0	1.0

**Fig. 3:**
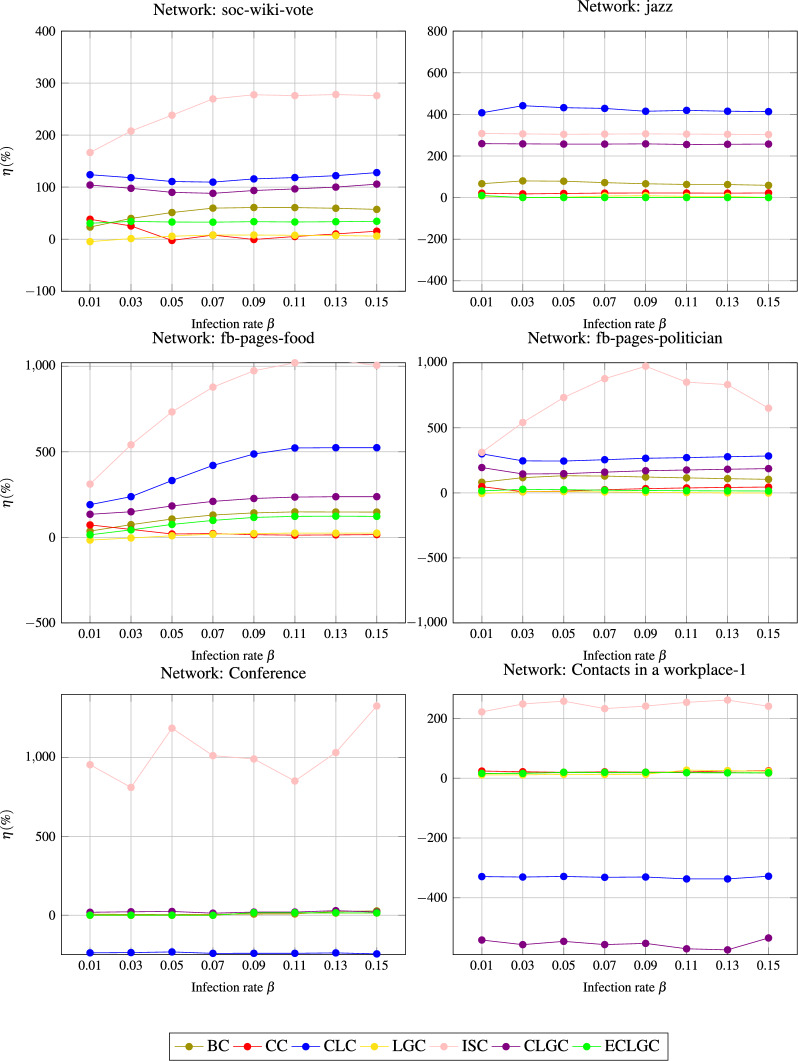
The percentage improvement $\eta \%$ of *EDDC* compared to the baseline indexing methods.

### Evaluating the percentage improvement $\eta \%$

This assesses the degree of improvement of the *EDDC* metric compared to the existing indexing methods. The performance gain metric ($\eta \%$)^54^ is denoted as:

15 $$\begin{aligned} \eta _\phi (\%)={\left\{ \begin{array}{ll} \frac{\tau _C(\phi ) -\tau _\phi }{\tau _\phi } * 100, & \tau _\phi >0\\ \frac{\tau _C(\phi ) -\tau _\phi }{-\tau _\phi } * 100, & \tau _\phi <0\\ 0, & \tau _\phi =0 \end{array}\right. } \end{aligned}$$

In this context, $\tau _C(\phi )$ denotes the Kendall $\tau$ between the *EDDC* and the SIR model, whereas $\tau _\phi$ represents the Kendall $\tau$ between the existing indexing metrics and the SIR model. When $\eta = 0$, $\eta > 0$, and $\eta < 0$, indicates that the proposed centrality measure performs similar, better, and worse to the baseline methods, respectively. Figure 3 shows the percentage improvement $\eta \%$ across the different complex networks analyzed. Across all the evaluated networks, the $\eta \%$ values for *C*(*BC*), *C*(*CC*), *C*(*CLC*), *C*(*LGC*), *C*(*ISC*), *C*(*CLGC*), and *C*(*ECLGC*) consistently show substantial improvement when $\beta > \beta _{th}$. Overall, the empirical results showed that the proposed *EDDC* measure performs better compared to the existing benchmark approaches in terms of $\eta \%$.

Furthermore, we have included the Top-10 node ranking distributions for the X and Y networks (refer to Table and Table 7) to demonstrate the effectiveness of EDDC compared to various indexing approaches. A significant overlap was found between the Top-10 nodes ranked by our method and those identified by existing algorithms, which reinforces the reliability and robustness of the proposed EDDC approach.

**Table 6:** Comparison of the Top-10 node rankings in the Jazz network across ten distinct approaches.

Rank	${\varvec{BC}}$	${\varvec{CC}}$	${\varvec{CLC}}$	${\varvec{LGC}}$	${\varvec{ISC}}$	${\varvec{CLGC}}$	${\varvec{ECLGC}}$	${\varvec{EDDC}}$
1	67	67	25	67	70	158	7	67
2	31	7	29	7	35	152	67	7
3	7	23	77	20	115	170	20	23
4	70	90	99	23	175	172	23	20
5	23	93	144	90	163	144	18	90
6	32	20	158	18	1	171	13	93
7	47	74	185	13	2	173	19	13
8	30	101	186	93	3	188	15	18
9	62	125	188	109	4	185	101	74
10	93	109	196	74	5	186	14	109

**Table 7:** Comparison of the Top-10 node rankings in the fb-pages-politician network across ten distinct approaches.

Rank	${\varvec{BC}}$	${\varvec{CC}}$	${\varvec{CLC}}$	${\varvec{LGC}}$	${\varvec{ISC}}$	${\varvec{CLGC}}$	${\varvec{ECLGC}}$	${\varvec{EDDC}}$
1	5800	5800	34	1864	3008	4167	1595	5800
2	1864	4081	42	5800	5800	830	5416	1864
3	3576	2059	43	4874	191	4944	1864	4081
4	2900	4032	62	4602	1864	5795	2386	4032
5	1324	3387	70	5416	4874	636	147	3576
6	3008	3576	73	3576	3099	5546	4602	4602
7	1965	1965	105	1595	3576	804	5867	2059
8	4395	4585	129	1474	4395	2588	1414	1474
9	191	2900	141	4032	5644	2005	4972	4874
10	1836	4622	145	4081	2218	1668	98	1965

### Time complexity analysis of EDDC

The EDDC method computes an influence score for each node by integrating degree, entropy, and shortest-path-based distance metrics. The main computational cost arises from calculating the shortest paths from each node to all others. To efficiently compute these distances, we use Dijkstra’s algorithm with a min-heap (priority queue). For a single node, Dijkstra’s algorithm runs in $O((N + E)\log N)$. Repeating this for each of the $N$ nodes (to compute all-pairs shortest paths) results in an overall complexity of $O(N(N + E)\log N)$. Compared to various traditional approaches, BC and CC exhibit a time complexity of $O(N^3)$. Therefore, we have conducted an experimental comparison of the time complexity of EDDC, BC, and CC by generating Erdõs-Rényi graphs^56^ with *N* nodes, where *N* ranges from 100 to 1000. Table 8 presents a comparison of the time complexity between BC, CC, and the proposed EDDC measure. It is important to acknowledge that there is often a trade-off between performance and computational complexity. In line with this, the proposed EDDC measure consistently demonstrates superior performance across all the considered networks when compared to traditional centrality measures. However, this improved performance comes at the cost of slightly higher time complexity relative to BC. To mitigate the computational load when applying EDDC to large-scale networks, practical optimizations can be employed. Notably, since Dijkstra’s algorithm computes the shortest paths from a single source to all other nodes in the network, the process can be computationally intensive. To reduce this burden, the computation can be restricted to nodes within a limited hop distance, such as 2 or 3 hops from the source node. This localized approach significantly reduces the number of shortest path calculations while still capturing meaningful topological information for the EDDC measure.

**Table 8:** Execution Time Comparison (in seconds) of EDDC, BC, and CC on Erdõs-Rényi networks.

Number of Nodes	EDDC Time (s)	BC Time (s)	CC Time (s)
100	0.03	0.02	0.01
200	0.16	0.12	0.02
300	0.49	0.32	0.04
400	2.08	1.09	0.08
500	2.14	1.32	0.12
600	5.51	2.14	0.17
700	7.83	3.28	0.26
800	10.87	5.90	0.61
900	14.97	8.28	0.85
1000	21.10	9.41	0.91

## Conclusions

The paper addresses the shortcomings of current state-of-the-art methods and newly suggested hybrid approaches by introducing a novel measure for finding prominent nodes in a variety of complex network configurations. The proposed approach demonstrates high efficiency in selecting influential nodes, yielding results closely aligned with the SIR model, as validated through Kendall’s correlation metrics. The EDDC approach addresses the shortcomings of the K-shell method by assigning a unique ranking to all nodes. Furthermore, using entropy as a local measure improves the accuracy of structural information derived from neighboring nodes. Unlike more recent methods such as CLGC and ECLGC, our approach demonstrates superior performance compared to the SIR model. All the experiments show significantly better results compared to the existing baseline approaches. In future work, our aim is to address the dynamic nature of networks by incorporating temporal information. To enhance the robustness of our methodology, future extensions could integrate complementary metrics such as the CLC (to capture neighborhood density), EC or PageRank (to account for recursive importance), or coreness (to identify nodes within cohesive subgroups). These could either support or replace the entropy term depending on the structural properties of the specific network under analysis. Additionally, we plan to expand this research by using deep learning techniques, such as studying graph neural networks, node embeddings etc.

## Data Availability

The proposed work used six publicly available complex networks datasets, namely, soc-wiki-vote, jazz, fb-pages-food, fb-pages-politician, Conference, and Contacts in a workplace-1. The datasets are downloaded from the following links: https://networkrepository.com/ and http://www.sociopatterns.org/datasets/.
